# Fabrication of Highly Microporous Structure Activated Carbon via Surface Modification with Sodium Hydroxide

**DOI:** 10.3390/polym13223954

**Published:** 2021-11-16

**Authors:** Mohd Sahfani Hafizuddin, Chuan Li Lee, Kit Ling Chin, Paik San H’ng, Pui San Khoo, Umer Rashid

**Affiliations:** 1 Institute of Tropical Forestry and Forest Products, Universiti Putra Malaysia, Serdang 43400, Malaysia; hafizsahfani@gmail.com (M.S.H.); kitling.chin419@gmail.com (K.L.C.); sansan_0928@hotmail.com (P.S.K.); 2 Faculty of Forestry and Environment, Universiti Putra Malaysia, Serdang 43400, Malaysia; 3 Institute of Nanoscience and Nanotechnology (ION2), Universiti Putra Malaysia, Serdang 43400, Malaysia; umer.rashid@yahoo.com

**Keywords:** concentration, coconut shell, palm kernel shell, surface area, adsorption, post-treatment

## Abstract

The aim of this study was to select the optimal conditions for the carbonization process followed by surface modification treatment with sodium hydroxide (NaOH) to obtain a highly microporous activated carbon structure derived from palm kernel shells (PKS) and coconut shells (CS). The effects of the carbonization temperature and NaOH concentration on the physiochemical properties, adsorption capability, specific surface area, surface morphology, and surface chemistry of PKS and CS were evaluated in this study. The results show that surface-modified activated carbons presented higher surface area values (CS: 356.87 m^2^ g^−1^, PKS: 427.64 m^2^ g^−1^), smaller pore size (CS: 2.24 nm, PKS: 1.99 nm), and larger pore volume (CS: 0.34 cm^3^ g^−1^, PKS: 0.30 cm^3^ g^−1^) than the untreated activated carbon, demonstrating that the NaOH surface modification was efficient enough to improve the surface characteristics of the activated carbon. Moreover, surface modification via 25% NaOH greatly increases the active functional group of activated carbon, thereby directly increasing the adsorption capability of activated carbon (CS: 527.44 mg g^−1^, PKS: 627.03 mg g^−1^). By applying the NaOH post-treatment as the ultimate surface modification technique to the activated carbon derived from PKS and CS, a highly microporous structure was produced.

## 1. Introduction

Malaysia is a country that is blessed with an abundance of water sources. However, 53% of the river water in Malaysia is classified as slightly polluted or polluted. River water pollution has recently become a serious problem and adversely affects the sustainability of water resources in the country. High levels of river water pollution have occurred in the states with large numbers of industrial zones and factories, including Selangor, Johor, Penang, and Perak. Industrial areas, sewage, agricultural activities, and animal husbandry activities are the three most common anthropogenic activities that cause river water contamination [1]. Thus, the issue of water treatment research is a critical priority because it has a direct impact on people’s lives, social development, and ecological health (i.e., natural, biological, and environmental) [2].

Chemical treatment is among the government’s efforts to overcome the issue of river water pollution [3,4]. Still, natural organic matter is hard to remove through chemical treatment [5]. Another efficient method for water treatment is the adsorption method, which is one of the most frequently considered treatments to be used in combination with chemical treatment to achieve the highest possible removal efficiencies of natural organic matter and enhance water treatment performance. Activated carbon (a universal adsorbent) is normally used in this adsorption method. With its large surface area and high adsorption capacity, activated carbon is commonly used to remove emerging contaminants from water and wastewater [6]. Dyes, heavy metals, petroleum hydrocarbons, pharmaceuticals, pesticides, and other organic matter can be absorbed by activated carbon [7]. However, due to fluctuating prices and the sustainability issues of coal, the widespread use of activated carbon is sometimes restricted [8]. Recently, considerable attention has been given to biomass conversion as a platform to economically produce activated carbon from biorenewable resources with quality that is comparable to coal-based activated carbon [9]. Hence, the conversion of biomass into activated carbon can provide an alternative way to reduce the carbon emissions, reuse waste materials, and contribute significantly to mitigating climate change [10]. Among the different types of biomass, the high density and rigidity of nutshell biomass such as coconut shells (CS) and palm kernel shells (PKS) make for a promising, modifiable material for highly microporous activated carbon production. Additionally, both of these lignocellulosic biomass are still underutilized in Malaysia. Thus, CS and PKS have the aptitude for use as inexpensive adsorbents, as they are not only an underused resource are easily available and are a sustainable resource [11].

Generally, biomass materials have inherent limits and have low adsorption capacity without additional treatment [12,13]. To surmount these limitations, processes such as amination, sulfonation, surface oxidation, and pore structure modifcation can provide new insights into the preparation of high-adsorption adsorbent materials [14]. Moreover, the development of micropores leads to the creation of high-surface areas [15]. Thus, in the present study, an attempt has been made to produce highly microporous activated carbon derived from CS and PKS to enhance their adsorption capacity via surface modification with sodium hydroxide (NaOH). NaOH presents three advantages in terms of lower weight dosage, lower cost, and the least amount corrosion compared to KOH. Furthermore, the by-products produced from NaOH activation, such as Na_2_CO_3_ and Na_2_SiO_3_, are non-toxic and non-hazardous. Both by-products (Na_2_CO_3_ and Na_2_SiO_3_ without any other contaminant) could be reused as raw materials after the filtration process [16]. Henceforth, a surface modification process using NaOH could be regarded as clean and green technology.

The overall objective of this work is to investigate the effects of NaOH surface modification (concentration of NaOH) on the physiochemical and surface characteristics of activated carbons derived from CS and PKS. The main limitations to the adsorption capacity that are extremely difficult to control in commercial carbons are poor pore size characteristics and surface properties [15]. Carbonization processes play a significant role in the development of the initial pore structure. The initial pores that are created during the carbonization process is an important basic feature of activated carbon that has a major influence on the following NaOH post-treatment effects on the characteristics of the final structure of the activated carbon. Thus, the aim of this study was to discover the ideal fabrication conditions for the carbonization process followed by NaOH post-treatment to obtain a highly microporous activated carbon structure derived from palm kernel shells (PKS) and coconut shells (CS). The relationships between the surface structure and the pore assessment were also evaluated. In order to study the effect of micropore characteristics on the surface area of the activated carbon, the data were analysed to examine the correlation between the micropore surface area and the total surface area.

## 2. Materials and Methods

### 2.1. Fabrication of Activated Carbon

CS was collected from Pasar Besar Kuantan, Pahang, and PKS was collected from Seri Ulu Langat Palm Oil Mill, Dengkil Selangor. Both types of nutshells were cleaned and dried in an oven at 105 °C for 48 h. The dried nutshells were then crushed and sieved to a 2–5 mm size range.

A specified mass of the dried nutshells were impregnated with 30% phosphoric acid (H_3_PO_4_) with an impregnation mass ratio of 1:1 at 80 °C for 2 h. The pretreated particles were filtered and washed with distilled water. The washed H_3_PO_4_ pretreated particles were then dried at 105 °C for 48 h prior to the carbonization process. The dried pretreated samples were then placed in the muffle furnace to undergo the carbonization process with relative temperatures of 500, 600, and 700 °C for 75 min. The activated carbons were kept in desiccators for further evaluation.

### 2.2. Surface Modification with NaOH

The specified mass of the activated carbon was soaked with NaOH (impregnation ratio of 1:1) with four different concentrations (6.25, 12.5, 25, and 50%) for 24 h. Finally, all of the obtained activated carbons were thoroughly washed with distilled water until they attained a pH of 6 to 7. The washed samples were dried at 105 °C for 48 h and were then stored in desiccators.

### 2.3. Evaluation

The characteristics of the activated carbon, including mass yield, ash content, methylene blue adsorption, iodine blue adsorption, BET surface areas, and surface characteristics were determined as previously described [10,13,14].

#### 2.3.1. Mass Yield

The activated carbon yield was calculated as below

(1) $$\mathrm{Yield}\;(\%)=\frac{\mathrm{Dry}\;\mathrm{weight}\;\mathrm{of}\;\mathrm{final}\;\mathrm{activated}\;\mathrm{carbon}\;\left(g\right)}{\mathrm{Dry}\;\mathrm{weight}\;\mathrm{of}\;\mathrm{raw}\;\mathrm{material}\;\left(g\right)}\;\times \;100$$

#### 2.3.2. Ash Content

The TAPPI standard method, T211 om-85, was used to determine the ash content. The oven-dried sample (2 g) was burnt (dry oxidation) in a muffle furnace model at 575 ± 25 °C for 4 h. This standard test method was used to determine the volume of ash remaining after the dry oxidation of the sample. The ash percentage was calculated by:

(2) $$\mathrm{Ash}\;(\%)=\frac{\mathrm{Weight}\;\mathrm{of}\;\mathrm{solids}\;\mathrm{remaining}\;\left(g\right)\;}{\mathrm{Original}\;\mathrm{weight}\;\mathrm{of}\;\mathrm{carbon}\;\left(g\right)}\times 100$$

#### 2.3.3. Methylene Blue Adsorption

The methylene blue number is defined as the milligrams of methylene blue or maximum amount of dye adsorbed on 1.0 g of adsorbent. The methylene blue number was determined according to the standard method (JIS K 1470-1991). In this assay, 0.05 g of adsorbent were placed in contact with 50 mL of a methylene blue solution at different concentrations (10, 25, 50, 100, 250, 500, and 1000 mgL^−1^) for 24 h at room temperature (of 25 °C ± 2 °C). After shaking for 24 h, the suspensions were filtered, and the remaining concentration of methylene blue in the solution was determined spectrophotometrically at a λ max of 660 nm using an UV/Vis spectrophotometer (UV- CECIL- CE-100). Standard methylene blue solutions were used for calibration. The amount of methylene blue adsorbed from each solution was calculated as below:

(3) $$\mathrm{Methylene}\;\mathrm{blue}\;\mathrm{adsorption}\;(\mathrm{mg}/g)=\frac{\left[\left(C0-\mathrm{Ce}\right)\;\times \;V\right]\;\left(\mathrm{mg}\right)\;}{M\;\left(g\right)}\times 100$$

where:

C0 (mg L^−1^) = the concentration of the methylene blue solution at starting time (t = 0);

Ce (mg L^−1^) = the concentration of the methylene blue solution at equilibrium time;

V (L) = the volume of the solution treated;

M (g) = the mass of the adsorbent.

#### 2.3.4. Iodine Adsorption Number

The ASTM D4607-94 method was used to determine the iodine adsorption number for the carbons. The iodine adsorption number can be explained as the milligrams of iodine adsorbed by 1.0 g of carbon. A conical flask with 10 mL of 5% HCl and 1.0 g of activated carbon was swirled until all of the activated carbon was wetted. The wetted activated carbon was boiled for exactly 30 s, and the solution was cooled to room temperature. An amount of 100 mL of 0.1 N (0.1 Mol L^−1^) iodine solution was then added to the mixture in the conical flask. The mixture was later filtered using a Whatman 2V filter paper. Finally, 50 mL of this filtrate was titrated with 0.1 N (0.1 Mol L^−1^) sodium thiosulphate in the presence of starch as indicator. The amount of iodine adsorbed per gram of carbon was calculated as shown in Equation (4):

(4) $$\mathrm{Iodine}\;\mathrm{adsorption},\;(\mathrm{mg}/g)=(\{(N_{1}\;\times \;126.93\;\times \;N_{2})\;-\;[(S_{1}\;+\;H_{1})/F_{1}]\;\times \;(S_{1}\;\times \;126.93)\;\times \;S_{2}\})/M$$

where:

N_1_ = Iodine solution normality;

N_2_ = Added volume of iodine solution;

H_1_ = Added volume of 5% HCI, ml;

F_1_ = Filtrate volume used in titration, ml;

S_1_ = Sodium thiosulfate solution normality;

S_2_ = Consumed volume of sodium thiosulfate solution, ml;

M = Mass of carbon, g.

#### 2.3.5. Surface Characteristic

The BET surface areas were determined by nitrogen adsorption at 77 K. Once the carbon was being degassed at 300 °C in an inert condition for 24 h, the nitrogen gas adsorption measurements were completed. A relative pressure of between 10.5 and 0.995 of nitrogen gas was used to obtain the N_2_ adsorption isotherm. Porosity is defined as the ratio of the total pore volume to the volume of the particle or agglomerate. In the context of physisorption, it is expedient to classify pores according to their size (IUPAC recommendation).

#### 2.3.6. Surface Morphology

The surface characteristics of a material refer to the properties associated with its surface. Typically, measurements of surface area, surface roughness, pore size, and reflectivity constitute surface characteristics. To obtain the micrographs of the prepared activated carbon, a scanning electron microscope was used. Scanning electron microscopy (SEM) analysis was conducted under optimum conditions to analyse the surface texture and the development of porosity in the activated carbon. In the SEM analysis, a small quantity of the dried samples was placed in the sample container of a scanning electron microscope and was sputtered with gold and palladium to acquire sufficient conductivity.

#### 2.3.7. FTIR

The surface functional groups of the samples were analysed using a Fourier transform infrared spectroscope (FTIR- Perkin Elmer Spectrum 100-IR, Tokyo, Japan). The dry samples were crushed into powder form and were inserted into the FTIR chamber. The spectra were recorded at a resoltion from 4000 to 700 cm^−1^ in the mid-infrared region.

### 2.4. Statistical Analysis

Statistical analyses were conducted using the statistical package SPSS for Windows, version 16.0 (SPSS, Chicago, IL, USA), which was used to evaluate the adsorption property data of the activated carbons for analysis of variance (ANOVA) at a 95% confident level (*p* ≤ 0.05). The Tukey–Kramer multiple comparisons test was applied to analyse the differences between the treatment effects when significance was observed. The effects were considered to be not statistically significant when the *p*-value was higher than 0.05 at the 95% confidence level. In this research, the Pearson correlation was used to develop a correlation between the proportion of the micropores and the specific surface area.

## 3. Results and Discussion

### 3.1. Physiochemical Properties of the Activated Carbon

The physiochemical properties of the activated carbon without NaOH surface modification (as shown in Table 1) were used as the reference samples in this study. According to the analysis of variance (ANOVA), the combination effects of the carbonization temperature and NaOH post-treatment concentration on the mass yield and ash content were highly significant (*p* < 0.01). The average values that were significant were compared using Tukey’s test and are summarized in Table 2.

Table 1 illustrates carbonization treatments for the CS and PKS at temperatures ranging from 500 to 700 °C. For both of the reference activated carbons, the mass yield decreases significantly while the ash content increases as the carbonization temperature increases. Table 2 presents the yield of the activated carbon obtained from the combination effects of the carbonization temperature and the NaOH post-treatment concentrations. As stated by Xu et al. [17], to balance the porosity and density of carbon, the carbonization temperature must be optimized. It was discovered that the yield of the activated carbons that were produced were inversely proportional to the carbonization temperature, with a lower yield obtained at higher carbonization temperatures. This could be due to the release of more volatile matter when carbon is activated at a higher temperature [18]. A lower activated carbon yield was obtained due to the release of a higher amount of volatile matter from the char [19]. A large number of gaseous components, including the surface oxygen (O)-containing groups, and contents of hydrogen (H), N, and O were greatly reduced when the temperature was raised from 500 °C to 900 °C [20,21]. The effects of the NaOH concentration on the activated carbon mass yield are shown in Table 2. An activated carbon mass yield of up to 36% was obtained from the PKS and CS carbonization via the approach including the addition of NaOH. From Table 2, it is evident that the mass yield increased by 11% and 17% when there were surface modifications using a NaOH concentration of 6.25% for CS and PKS activated carbon, respectively. A further increase in the concentration of NaOH resulted in a steady reduction in the mass yield. This indicates that the application of a solution with a greater number of alkali metal hydroxides facilitated the removal of more volatile components from the char during the subsequent surface modification process. In addition, more alkaline hydroxide molecules were used to break chemical bonds such as alkyl–aryl bonding within the char matrix when higher chemical concentrations were used [19]. In addition, NaOH provided an elimination and dehydration reaction, breaking the C–O–C and C–C bonds of the raw material, also justifying a decline in carbon yield [22].

High ash content in agricultural biomass such as PKS and CS is one of the main barriers to achieving high-quality activated carbon [15]. The positive effects of the NaOH surface modifications were counteracted by the amount of ash left on the activated carbon. Table 2 reveals that NaOH surface modification can potentially lower the ash content in activated carbon to 24%. The alkali used in this process is expected to react the mineral constituents as follows:

2NaOH + SiO_2_ → Na_2_SiO_3_ + H_2_O (5)

2NaOH + AlO_2_O_3_ → 2NaAlO_2_ + H_2_O (6)

As shown in reaction Equations (5) and (6), the removal of the major part of the quartz and alumina is affected by the formation of sodium silicate and aluminate [23]. When the NaOH concentration increased, the ash content increased. Furthermore, higher concentrations do not appear to be economically feasible for industrial-scale demineralization. Therefore, it was preferable to create a microporous active carbon structure with the lowest concentration of NaOH (6.25–25%) possible. Table 2 shows that the ash content of the activated carbon increased as the carbonization temperature increased. When the temperature was raised from 500 to 700 °C, the ash content in the CS and PKS increased from 24.16 to 26.30% and 24.10 to 26.01%, respectively. The increase in ash content is caused by the progressive concentration of minerals and the destructive volatilization of lignocellulosic materials as the temperature rises [18].

### 3.2. Adsorption Properties of the Activated Carbon

The adsorption properties of the surface-modified activated carbon derived from CS and PKS are compiled in Table 3. The ANOVA analysis revealed the combination effects of the carbonization temperature and the NaOH post-treatment concentration on the methylene blue and iodine adsorption properties were highly significant (*p* < 0.01). The average values that were significant were compared using Tukey’s test and are summarized in Table 3.

According to Chin et al. [15], methylene blue is used to measure pores greater than 1.5 nm in diameter. The combination effects of carbonization temperatures and NaOH post-treatment concentrations on methylene blue adsorption are shown in Table 2. An increase in the adsorption properties was observed with the application of a surface modification technique using NaOH. The lower adsorption rate could be due to a scarcity of active sites on the activated carbon’s surface. As the porosity of the adsorbent increases, the number of active sites available for methylene blue adsorption increases [11]. Aside from that, the increasing of the adsorption properties with the increasing of NaOH concentration could be due to the removal of ash components from the activated carbon. The adsorptive nature of the activated carbon was questioned due to the amount of ash content that was present because it is not only causes an inhibiting effect but also affects the adsorption characteristic of different organic chemicals. However, increasing the concentration of NaOH to 25% reduced the adsorption of methylene blue on both types of the activated carbons. The result indicates that the use of high carbonization temperatures accelerates the NaOH surface modification process. Table 3 depicts how the surface-modified samples from the activated carbon prepared at 700 °C achieved the highest adsorption capability. This may be due to activated carbon prepared with high temperatures and with a low moisture content, which can provide more adsorption sites becoming available for use as an adsorbent. The moisture content in the activated carbon would have blocked the adsorption sites of the activated carbon, thus decreasing its adsorption efficiency [19]. Moreover, thermal treatment resulted in a higher rate of volatile content removal and pore structure development [15].

Iodine adsorption is the most fundamental parameter used to define and characterize the performance of an adsorbent [24]. The iodine value can indicate the total surface area and micropore volume of activated carbon [11]. According to Chin et al. [15], the discovery of new methods for controlling porosity and microarchitecture has the potential to lead to the refinement of lignocellulose carbon resources as advanced functional materials. Thus, NaOH surface modification has a high potential to produce a highly microporous activated carbon structure that can be used to remove a variety of pollutants from wastewater effluent. When CS activated carbon was modified with 25% NaOH, it exhibited the highest iodine adsorption. The increased iodine adsorption capacity is a result of the high degree of activation. Activated carbon prepared via 50% NaOH had the lowest iodine adsorption. The micropores might undergo coalescence as a result of deeper carbon decomposition where there is a higher NaOH concentration.

Table 1 illustrates that the carbonization temperature is critical in the pre-formation of pores (reference activated carbon) in order to produce highly microstructured activated carbon. This could be due to the fact that some volatile compounds can only be removed at higher carbonization temperatures (>650 °C) [18]. Higher volatile compounds in the reference sample may “block” pore spaces, resulting in a smaller surface area. Simultaneously, increasing the carbonization temperature from 500 °C to 700 °C prior to surface modification treatment resulted in an increased of iodine adsorption for the surface-modified samples. This may be attributable to the reaction of NaOH and the development of new pores in the carbon structure [25]. The chemical reactions of NaOH and carbon during the surface modification process can be written as follows [26]:

4NaOH + C → Na_2_CO_3_ + Na_2_O + 2H_2_ (7)

2Na_2_O + C → 4Na + CO_2_ (8)

Na_2_CO_3_ + 2C → 2Na + 3CO (9)

Based on these findings, it was ascertained that the pre-formation of porous entities on the surface structure of the activated carbon facilitated the NaOH surface modification process. Moreover, alkali materials are well known for being able to penetrate swollen cellulose fiber and to increase certain areas of the cell wall, making chemical interactions easier. After alkali treatment, lignin was removed extensively, the mechanical properties of the cell wall were decreased, the cellulose fibrils were collapsed, and the porosity of the surface was increased [27]. This explains how NaOH surface modification on activated carbon prepared at 700 °C achieved the highest iodine adsorption.

### 3.3. Surface Characteristic of Activated Carbon

**Figure 1:**
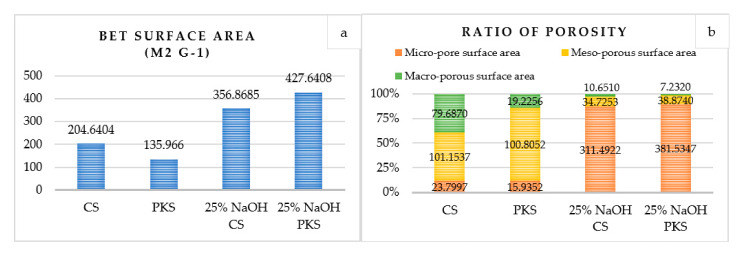
Surface characteristics of activated carbon: (**a**) BET surface area and (**b**) porosity ratio for activated carbon prepared at 700 °C for 75 min (reference sample) and activated carbon (700 °C, 75 min) post-treated with 25% NaOH.

**Figure 2:**
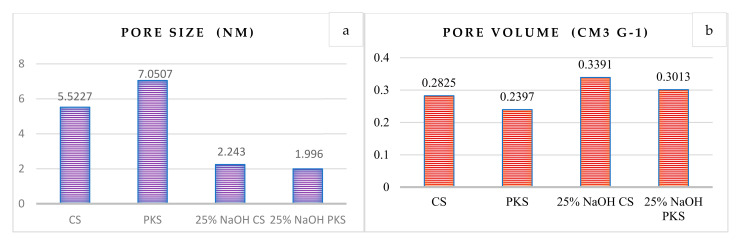
Pore measurements of activated carbon: (**a**) Pore size and (**b**) pore volumes for activated carbon prepared at 700 °C for 75 min (reference sample) and activated carbon (700 °C, 75 min) post-treated with 25% NaOH.

In this study, the reference samples and surface-modified activated carbon with the highest adsorption properties were evaluated on the surface area. This includes reference-activated carbon samples prepared at 700 °C for 75 min and surface-modified activated carbon (700 °C, 75 min) post-treated with 25% NaOH. Figure 1 depicts the total BET surface area and porosity ratio of the prepared activated carbon samples, while Figure 2 illustrates pore measurements (pore size and pore volume) of the samples.

According to the porosity ratio (Figure 1b), higher NaOH concentrations facilitate the production of activated carbon micropores. Micropores occupied 80% of the total pore ratio of the surface-modified activated carbon. Changes in the structure and adsorption properties of activated carbon were observed as a result of the surface modifications with NaOH. The following equations outline the proposed reactions during NaOH activation:

6NaOH + 2C → 2Na + 2Na_2_CO_3_ + 3H_2_ (10)

Na_2_CO_3_ → Na_2_O + CO_2_ (11)

2Na + CO_2_ → Na_2_O + CO (12)

The possible reactions between active substances and the surface of the organic precursor resulted in the formation of micropores on the activated carbon surface due to the release of CO, CO_2_, and H_2_ gases (refer to Equations (10)–(12)), which are produced by Na_2_CO_3_ decomposition at high temperatures and during hydroxyl reduction, respectively. Moreover, alkali metal intercalation into the carbon structure may result in the formation of an activated carbon micropore [28]. When the surface of PKS-activated carbon was modified with 25% NaOH, it achieved the highest surface area (427.64 m^2^/g), which was approximately three times greater than the activated carbon prepared without surface modification. This demonstrates that NaOH is critical for the development of a high surface area for activated carbon. It was also observed that the addition of NaOH increased the BET surface area but decreased the activated carbon yields. Figure 2 depicts the pore size distributions of the prepared activated carbon. The pore measurement results showed that the addition of NaOH resulted in a higher surface area for activated carbon by reducing the pore size. For the activated carbons prepared without surface modification using NaOH, the large pore size resulted in the least efficient adsorption properties. Furthermore, the high surface area could be attributed to the larger total pore volume in the surface modified activated carbons (refer Figure 2b).

**Figure 3:**
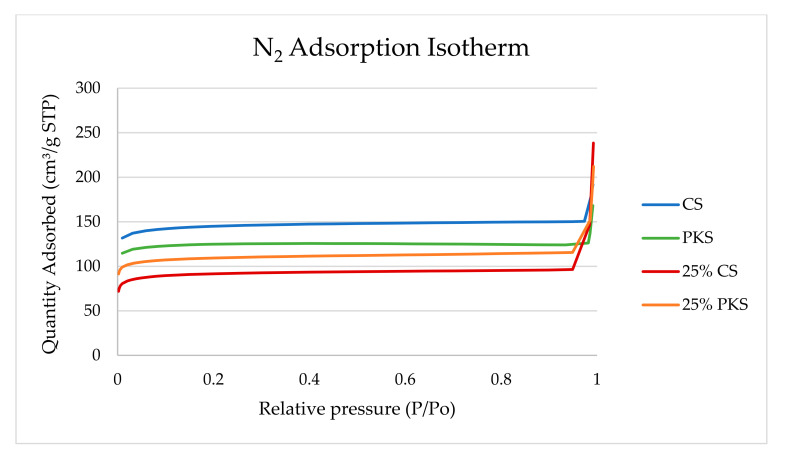
N_2_ adsorption isotherm for activated carbon prepared at 700 °C for 75 min (reference sample) and activated carbon (700 °C, 75 min) post-treated with 25% NaOH.

The N_2_ adsorption isotherm acquired from the prepared activated carbons is established in Figure 3. Both of the activated carbon reference samples prepared without surface modification conform to a type IV isotherm. Figure 3 signifies that the initial part of the type IV isotherm for carbon represents micropore filling, and the slope of the plateau at high relative pressure is due to multilayer adsorption on non-microporous surfaces, i.e., in mesopores, in the macropores, and on the external surface [29]. This infers that both of the activated carbon reference samples had a mesoporous structure that mostly contained mesopores. The activated carbons with NaOH surface modification conformed to the type I isotherm, disclosing that the activated carbons are produced by microporous solids with relatively small external surfaces such as activated carbons, molecular sieve zeolites, and certain porous oxides. The steep uptake at a very low p/p° is due to the enhanced adsorbent–adsorptive interactions in the narrow micropores (micropores of molecular dimensions), resulting in micropore filling at a very low p/p°. The type I isotherm implies the near absence of mesopores and macropores inside of the material [11]. The results show that the N_2_ adsorption isotherm for activated carbon correlates with the pore measurement results. For activated carbon with NaOH surface modification, the presence of micropores is advantageous.

### 3.4. Surface Morphology of Activated Carbon

Scanning electron micrography was performed on the reference samples (activated carbon without surface modification) and activated carbon with NaOH surface modification, which obtained the highest adsorption properties. This includes the reference samples (activated carbon prepared at 700 °C for 75 min) and the activated carbon (700 °C, 75 min) post-treated with 25% NaOH.

**Figure 4:**
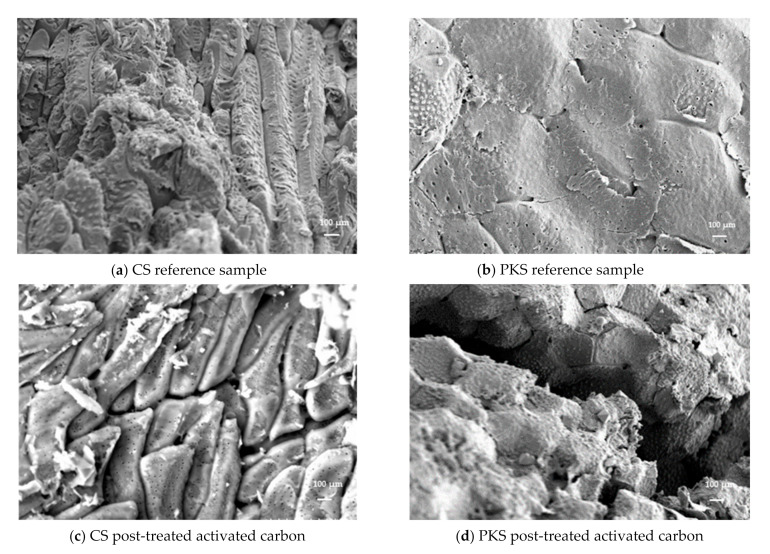
Scanning electron micrographs of (**a**,**b**) activated carbon prepared at 700 °C for 75 min and (**c**,**d**) activated carbon (700 °C, 75 min) post-treated with 25% NaOH at 1000× magnification.

This experiment further manifests the capability of NaOH surface modification to penetrate deeper into the structure of activated carbon, creating cavities and pores, thus increasing the surface area. The morphology analysis revealed that the reference samples have a poor pore structure. Figure 4a,b show that the reference samples almost have no microporous structure when compared to the activated carbon with NaOH surface modification. The pores of the CS reference samples were mostly closed, and the pores for the PKS reference samples were not visible during the SEM magnification, while surface modification with NaOH produced rugged surfaces with microporous properties on both types of nutshell-derived activated carbons, indicating that the porous structure is well developed. The surface modification technique used in this study mainly alters the pore structure of activated carbon, such as specific surface area, pore volume, and pore size. Furthermore, unclogged cavities were observed on the surfaces of both the activated carbon samples with NaOH surface modification (refer to Figure 4c,d). This clearly indicates that under the same carbonization temperature, the formation of microporous structures on the activated carbon were mainly contributed to by the additional NaOH surface modification stage. The surface morphology and surface characteristic analysis were used to explain the activated carbon’s high adsorption performance. Overall, a surface-activated carbon (surface modification with 25% NaOH) with excellent adsorption performance on iodine and methylene blue was obtained in this study.

### 3.5. Surface Chemical Characteristics of Activated Carbon

Evaluations of the surface chemical characteristics in this study were only performed on the activated carbon that obtained the highest adsorption properties and the highest surface area. This includes the reference samples (activated carbon prepared at 700 °C for 75 min) and activated carbon (700 °C, 75 min) post-treated with 25% NaOH.

**Figure 5:**
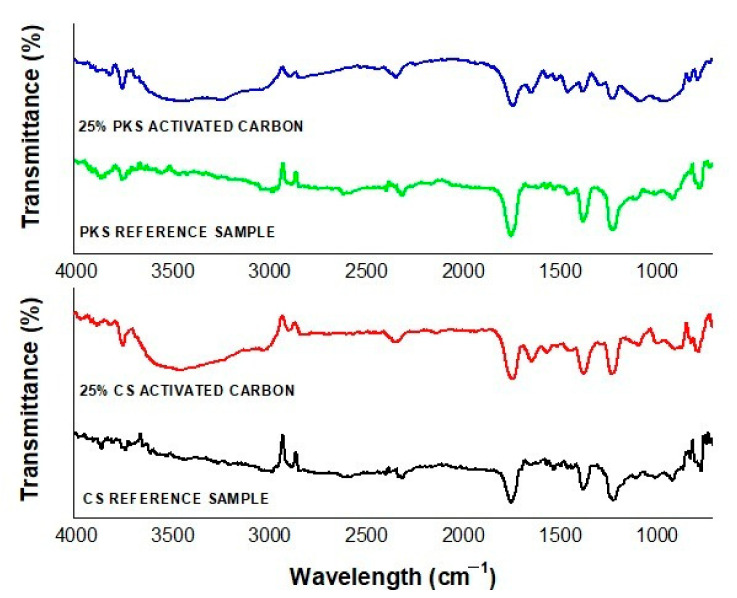
FTIR for CS and PKS activated carbons prepared in 700 °C for 75 min (reference sample) and activated carbon (700 °C, 75 min) post-treated with 25% of NaOH.

FTIR techniques were used to examine the functional and surface chemistry of the prepared activated carbon samples. As illustrated in Figure 5, activated carbon prepared without surface modification exhibits its characteristic peaks at 3438 cm^−1^, which are assigned to the hydroxyl (O-H). The strong peaks at 3455 cm^−1^ for activated carbon modified with 25% NaOH can be attributed to O-H stretching. The peak shift from 3438 cm^−1^ to 3455 cm^−1^ indicates that as a reducing agent, NaOH prevents hydrogen bonding and removes the OH functional groups on activated carbon [30]. Furthermore, the peaks corresponding to the O-H groups and the aliphite groups became weaker or disappeared with surface modification. Hence, the broad O-H peak was replaced by a sharp signal at 3455 cm^−1^. Carboxylic functional groups were observed in 2337 cm^−1^ [31,32] for the PKS-activated carbon modified with 25% NaOH. Moreover, the peaks at 3740 cm^−1^ and 3400 cm^−1^ corresponding to the -NH_2_ stretching and -OH stretching of hydroxyl groups [33] were observed in both of the NaOH surface modified samples. CS-activated carbon modified with NaOH caused the appearance of several new absorption peaks. These peaks can be classified as follows: C≡C group (2348 cm^−1^) [33] and C−N stretching vibration absorption peaks (1221 cm^−1^) [34]. Alternative polymerization conditions occurred with NaOH surface modification, which serves as a useful control to demonstrate the importance of surface area for rapid micropollutant removal [35]. The surface modification of activated carbon improves chemical adsorption by increasing the active functional groups on the surface [36]. This concludes that surface modification with NaOH greatly increases the functional group of activated carbon and thereby directly increases the adsorption capability of activated carbon.

### 3.6. Correlation of Surface Area and Micropore

**Figure 6:**
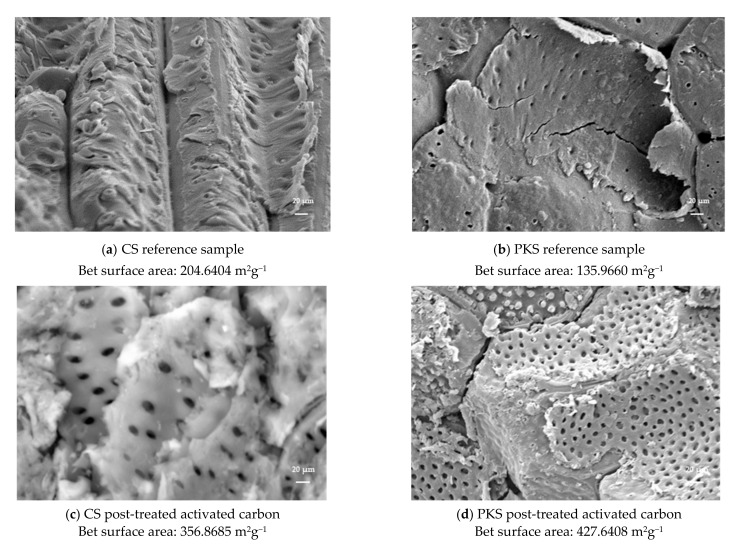
Scanning electron micrographs of (**a**,**b**) activated carbons prepared at 700 °C for 75 min and (**c**,**d**) activated carbon (700 °C, 75 min) post-treated with 25% NaOH at 2000× magnification.

In order to achieve the greatest possible activated carbon production from biomass, a precise understanding of the relationship between the surface structure and the pore assessment is required. Surface area and pore size are widely used parameters for activated carbon testing due to their simplicity and the availability of a rapid assessment for sample simplicity and a rapid assessment of the absorbent quality of the samples in question. The correlation results of the surface area and micropore surface area of the prepared activated carbon is given in Table 4, while Figure 6 presents the reference samples prepared at 700 °C for 75 min and activated carbon (700 °C, 75 min) post-treated with 25% NaOH at low magnification. Lower magnifications, which represent wider fields of view, allow for a broad range of pore sizes to be observed, though only larger pores can be identified. When the magnification is increased to a certain level, the field of view narrows and only a portion of the micropores is visible [37]. Thus, the different pore size distribution characteristics under different magnifications (Figure 4: 1000×; Figure 6: 2000×) demonstrate the importance of magnification in image analyses for the activated carbon.

Carbon pores have a wide range of sizes (widths) from 0.1 nm to more than 10 mm, which can be classified as micropores (less than 2 nm in size), mesopores (2–50 nm), and macropores (more than 50 nm). Micropores are further divided into ultramicropores (less than 0.7 nm in size) and supermicropores (0.7–2 nm) [38,39]. In this work, we tried to establish a correlation between the BET surface area and the microporosity of the activated carbon. The Pearson correlation coefficient is a measure of the linear relationship between two variables and is the most frequently used measure of association between variables [40]. The results (Table 4) show the strong significant correlation between the micropores on the specific surface area of the activated carbon derived from PKS and CS. In principle, the higher the proportion of microporosity, the greater the specific surface area. The growth at the specific surface area of activated carbon achieved with the presence of high a proportion of microporosity is also demonstrated in Figure 6.

Low magnification was applied in order to obtain the apparent pore structure on the surface of the activated carbon samples. Figure 6a,b shows that the surface structure of the reference sample was primitively rough with no visible pores. The SEM analysis suggested that NaOH surface modification was effective to fabricate activated carbon with a higher specific surface area and narrower size distribution. The SEM results (Figure 6c,d) show the presence of micropores in both samples prepared via NaOH surface modification, with the quantity of the pores being slightly increased with the decrease of the pore size. The PKS-activated carbon post-treated with NaOH (Figure 6d) reveals a large proportion of supermicropores, i.e., voids of widths less than 2 nm, while the untreated activated carbons (Figure 6a,b) possessed a bigger pore size and a larger proportion of mesopores. Principally, the specific surface area of activated carbon seems to be highly relevant with the proportion of microporosity. The results of the present study demonstrate that highly microporous activated carbon with a high surface area can be prepared from CS and PKS prepared with NaOH surface modification. Through the surface area characteristic and surface morphology results, micropores were found to be one of the most important elements in the preparation of a high grade activated carbon.

## 4. Conclusions

In this study, NaOH surface modification was introduced to fabricate microporous structure activated carbons. The results show that after surface modification, activated carbon has high surface and pore volume values, indicating that NaOH treatment is effective in improving the performance of nutshell-derived activated carbons. The entire 80% of the total pore ratio was occupied with micropores for both types of nutshell-derived activated carbons prepared via NaOH surface modification. This unique method is capable of creating new micropores on activated carbon. Surface modifications also improve adsorption capacity by increasing the active functional groups on the surface of carbon. The findings also show that using high carbonization temperatures accelerates the NaOH surface modification process. The highest adsorption capacity was achieved by NaOH-surface modified samples from activated carbon prepared at 700 °C. Furthermore, this study also discloses that a direct correlation between the proportion of micropores and the specific surface area is evident. A higher proportion of micropores in the carbon particles is associated with a higher specific surface area. By applying the NaOH post-treatment as the ultimate surface modification technique to the activated carbon derived from PKS and CS, a highly microporous structure was produced.

## Figures and Tables

**Table 1:** Physiochemical properties of activated carbon without NaOH surface modification.

Carbonization Temperature (°C)	Mass Yield (%)		Ash Content (%)		Methylene Blue (mg/g)		Iodine Adsorption (mg/g)	
	CS	PKS	CS	PKS	CS	PKS	CS	PKS
500	36.23	37.08	24.34	24.23	86.128	115.350	308.693	305.901
600	26.93	27.04	35.51	34.39	264.792	212.757	406.430	372.920
700	27.44	27.26	35.50	31.08	280.429	212.757	441.800	402.710

**Table 2:** ANOVA for the mass yield and ash content of activated carbon.

Carbonization Temperature (°C)	NaOH Concentration (%)	Mass Yield (%)		Ash Content (%)	
		CS	PKS	CS	PKS
500	6.25	36.19 ^a^	36.84 ^a^	24.16 ^a^	24.10 ^a^
500	12.5	35.42 ^b^	35.54 ^b^	25.31 ^b^	25.30 ^b^
500	25	33.75 ^e^	33.92 ^d^	27.01 ^d^	26.72 ^d^
500	50	32.82 ^f^	33.39 ^e^	27.20 ^e^	27.30 ^f^
600	6.25	35.31 ^c^	34.84 ^c^	26.30 ^c^	25.25 ^b^
600	12.5	34.53 ^b^	33.93 ^d^	27.01 ^d^	27.09 ^e^
600	25	28.12 ^i^	33.11 ^f^	29.51 ^g^	28.12 ^g^
600	50	27.63 ^j^	32.08 ^g^	31.10 ^i^	28.90 ^h^
700	6.25	30.91 ^g^	33.2 ^e f^	26.30 ^c^	26.01 ^c^
700	12.5	29.96 ^h^	31.08 ^h^	28.38 ^f^	29.50 ^i^
700	25	27.36 ^k^	29.65 ^i^	30.81 ^h^	31.16 ^j^
700	50	27.02 ^l^	28.20 ^j^	32.01 ^j^	32.49 ^k^
*p*-value		<0.001	<0.001	<0.001	<0.001

Note: Means followed by the same letter in the same column are not significantly different at *p* ≤ 0.05 according to Tukey’s multiple comparisons test.

**Table 3:** ANOVA for adsorption properties of activated carbon.

Carbonization Temperature (°C)	NaOH Concentration (%)	Methylene Blue (mg/g)		Iodine Adsorption (mg/g)	
		CS	PKS	CS	PKS
500	6.25	108.685 ^j^	173.025 ^j^	327.310 ^i^	314.279 ^k^
500	12.5	208.399 ^g^	208.912 ^g h^	336.618 ^h^	333.826 ^i^
500	25	187.380 ^h^	192.763 ^h i^	358.958 ^j^	353.373 ^h^
500	50	177.126 ^i^	185.842 ^h i^	309.625 ^j^	375.713 ^g^
600	6.25	350.408 ^d^	302.217 ^d^	307.763 ^j^	476.241 ^f^
600	12.5	400.905 ^a^	331.952 ^c^	414.807 ^e^	504.166 ^c^
600	25	395.266 ^b^	261.204 ^e^	442.732 ^d^	543.260 ^b^
600	50	388.345 ^c^	218.909f ^g^	361.751 ^g^	318.002 ^j^
700	6.25	296.321 ^e^	238.903 ^e f^	479.965 ^c^	487.411 ^e^
700	12.5	226.855 ^f^	409.877 ^a^	499.512 ^b^	543.260 ^b^
700	25	110.480 ^j^	376.297 ^b^	527.436 ^a^	627.034 ^a^
700	50	72.799 ^k^	257.871 ^e^	401.776 ^f^	495.789 ^d^
*p*-value		<0.001	<0.001	<0.001	<0.001

Note: Means followed by the same letter (a–k) in the same column are not significantly different at *p* ≤ 0.05 according to Tukey’s multiple comparisons test.

**Table 4:** Correlation of surface area and micropore of the prepared activated carbons.

Correlation		Micropore Surface Area	Total Surface Area
Micropore surface area	Pearson Correlation	1	0.858 **
	Sig. (2-tailed)		0.000
	N	18	18
Total surface area	Pearson Correlation	0.858 **	1
	Sig. (2-tailed)	0.000	
	N	18	18

** Correlation is significant at the 0.01 level (2-tailed).

## Data Availability

All relevant data are within the manuscript.
